# Lab-based feasibility and acceptability of neuromuscular electrical stimulation in hip osteoarthritis rehabilitation

**DOI:** 10.1177/2055668320980613

**Published:** 2021-03-16

**Authors:** Louise C Burgess, Paul Taylor, Thomas W Wainwright, Ian D Swain

**Affiliations:** 1 Orthopaedic Research Institute, Bournemouth University, Bournemouth, UK; 2 Department Clinical Science and Engineering, Salisbury District Hospital, Salisbury, UK; 3 Odstock Medical Limited, Salisbury District Hospital, Salisbury, UK; 4 Faculty of Health and Social Science, Bournemouth University, Bournemouth, UK; 5 Physiotherapy Department, The Royal Bournemouth Hospital, Bournemouth, UK

**Keywords:** Rehabilitation devices, rehabilitation, electrical stimulation, hip osteoarthritis, NMES

## Abstract

**Introduction:**

Neuromuscular electrical stimulation (NMES) could provide an alternative or adjunct treatment modality to induce muscle hypertrophy in the hip osteoarthritis population. This preliminary study evaluates the feasibility and acceptability of NMES to evoke involuntary muscle contractions in adults with advanced hip osteoarthritis.

**Methods:**

Thirteen adults with moderate-to-severe hip osteoarthritis and fifteen healthy, older adults were invited to a lab-based testing session. NMES was applied unilaterally to the knee extensors and hip abductors for one continuous, five-minute testing session. Data were collected on device acceptability, tolerability and muscle contractile force, and compared between groups.

**Results:**

Electrical stimulation of the knee extensors elicited a visible muscular contraction in 11 participants (85%) with hip osteoarthritis and 15 controls (100%) at an intensity acceptable to the participant. Electrical stimulation of the hip abductors elicited a muscular contraction in eight participants (62%) with osteoarthritis, and ten controls (67%). Muscle contractile force, pain, discomfort and acceptability did not differ between groups, however NMES of the knee extensors was favoured across all measures of assessment when compared to the hip abductors.

**Conclusions:**

Electrical stimulation of the knee extensors may be a feasible and acceptable treatment modality to address muscle atrophy in adults with advanced hip osteoarthritis.

## Background

Bilateral lower-limb muscle weakness and fatigue are features of individuals with advanced hip osteoarthritis,^1–6^ which can lead to functional disability and an increased risk of further morbidity and mortality.^7,8^ To counteract musculoskeletal impairment, local muscle strengthening and aerobic exercise are recommended irrespective of age, comorbidity, pain severity or disability.^9–12^ Likewise, when progression of the disease leads to consideration for total hip replacement surgery, preoperative exercise programmes are proposed as a potential method to expedite recovery time.^13–15^ Nonetheless, some patients choose to avoid traditional exercise due to fear of causing joint damage or exacerbating pain,^16–20^ and the evidence supporting physiotherapy prior to hip replacement for improving function is equivocal.^13^

Neuromuscular electrical stimulation (NMES) is an alternative treatment that can counteract muscle weakness in adults with advanced progressive diseases; and has long been used to preserve or restore skeletal muscle mass and function during and after a period of disuse due to injury, surgery, or illness.^21–23^ NMES involves the application of electrical impulses to skeletal muscles, by means of surface electrodes placed over the muscle belly, with the ultimate goal to evoke visible muscular contractions.^22^ The activation pattern of these contractions differs substantially from that of voluntary contractions, whereby motor units are recruited in a non-selective, spatially fixed, and temporally synchronous pattern.^24^ Whilst the force contracted through muscle stimulation is not greater than that of voluntary isometric contractions, it can be used where the pathology prevents voluntary exercise at either sufficient intensity or duration to be effective, with the end goal of moving onto voluntary exercise when strength and tolerance permits.^25–27^ In addition, it can be used as an adjunct modality to enhance the strengthening effects of an existing rehabilitation programme, or support patients with muscle weakness who cannot tolerate high-intensity exercise or a high-volume of low-intensity exercise.^21^

Despite the evidence supporting electrical stimulation as a method to improve muscle strength, voluntary activation and functional recovery, NMES therapy remains clinically underutilised in orthopaedic practice.^22,28,29^ Moreover, whilst there has been an expansion of research in the area of knee osteoarthritis and NMES for strength improvements, investigations within hip osteoarthritis are sparse.^23,30^ NMES may offer a promising alternative approach to counteract muscle inhibition and minimise atrophy and thus restore normal muscle function more effectively than voluntary exercise alone. This preliminary study aims to investigate the feasibility and patient acceptability of using NMES as a treatment option to counteract muscle weakness amongst adults with advanced hip osteoarthritis. Data are compared to healthy adults, to observe any differences in response to NMES that may be attributable to hip joint pathology.

## Methods

### Participants

This is an observational case-control study recruiting two study groups: i) adults with a clinical diagnosis of unilateral or bilateral hip osteoarthritis and ii) healthy adults aged over 60 years (control group) between 12th November 2019 and 15th March 2020. Participants were recruited from the local area through online advertisement and email recruitment sent to local organisations. Sixty years was chosen as the minimum age for the control group as osteoarthritis of the hip increases between the ages of 45 and 75,^31^ and the average age for total hip replacement surgery is 68.0 ± 11.4 years.^32^ Participants were included in the hip osteoarthritis group if they had: i) received a clinical diagnosis of hip osteoarthritis from their general practitioner, an orthopaedic specialist or a physiotherapist; ii) presented with chronic joint pain for at least three months; iii) had an Oxford Hip score^33^ of less than 40; and iv) were not on the waiting list for total hip replacement surgery. Participants were included in the control group if they were over 60 years old with no significant musculoskeletal comorbidities or neurological diseases. Exclusion criterion for both groups included: i) neurological disease affecting walking ability; ii) rheumatoid arthritis; iii) fitted with a pacemaker or other active medical implant; iv) uncontrolled epilepsy; v) sepsis or osteomyelitis; vi) known metastatic tumour involving the hip; vii) poor skin condition that prevented the use of self-adhesive electrodes; viii) not physically able to complete the testing protocol or ix) not able to provide informed consent. The experimental protocol was approved by the institutional ethics committee on 5th September 2019. In keeping with good practice, the ethical principles for medical research outlined in the Declaration of Helsinki were followed.^34^ The STROBE (Strengthening the Reporting of Observational Studies in Epidemiology) statement for the reporting of cross-sectional studies was used to guide the reporting of this study.^35^

### Electrical muscle stimulation device

The NMES device chosen for this study was the Orthopaedic Microstim 2V2 neuromuscular stimulator (developed by Odstock Medical Ltd, Salisbury, UK). The device has been developed for general orthopaedic use, and for following joint replacement surgery, and consists of a stimulator box with two leads which are connected to two multiple use self-adhesive electrodes. It includes specific programmes to target muscle conditioning, endurance or power, in addition to programmes aimed at improving venous return and preventing thrombosis and pain relief modes. The programme chosen for this study was mode 0 (“set-up”) which is most appropriate when first evaluating electrode positioning and stimulation intensity. Whilst it is more common for an intermittent stimulation to be delivered within clinical practice, this mode delivers a continuous stimulation output, which is useful for determining individual responses to NMES with a controlled approach. The mode delivers a frequency of 40 Hz and a pulse duration of 300µs.

### Procedures

**Figure 1.**
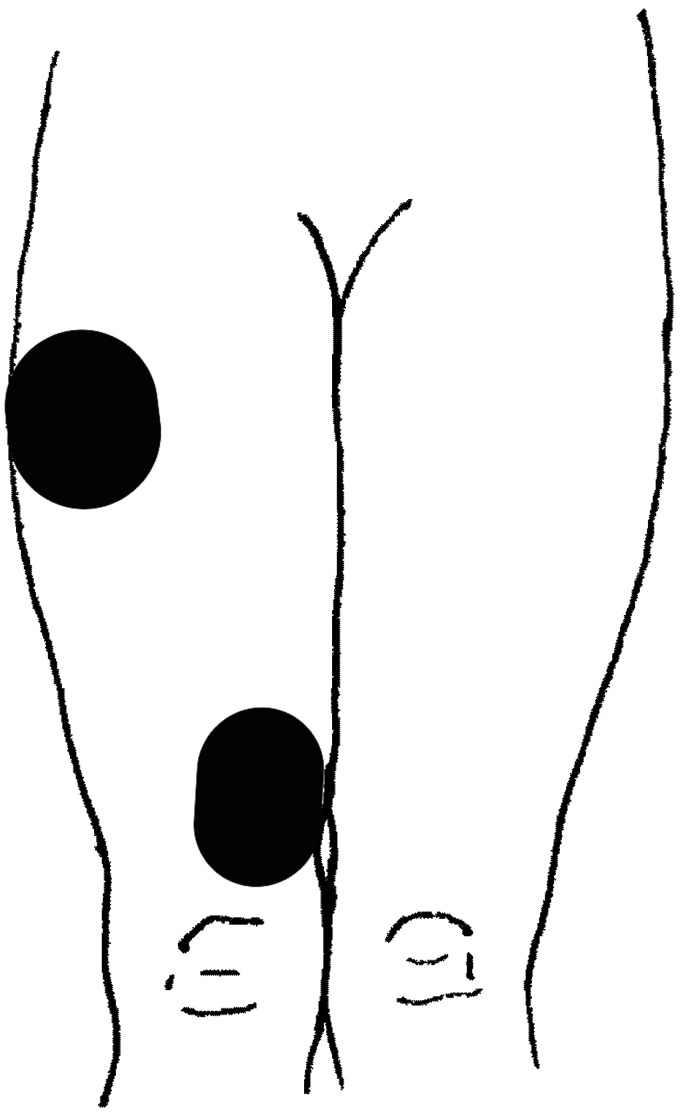
Electrode positioning during electrical stimulation of the knee extensors.

**Figure 2.**
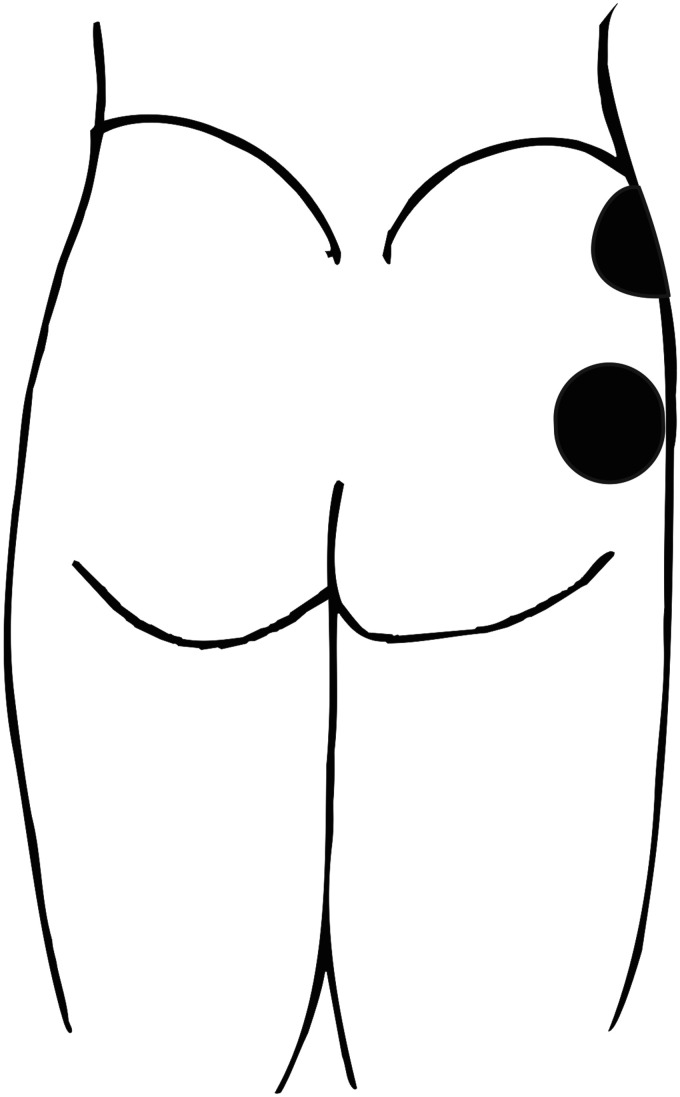
Electrode positioning during electrical stimulation of the hip abductors.

Participants were invited to attend a laboratory-based testing session. Participants were shown the NMES device and given instructions on how to operate it. The device was fitted by a researcher to the knee extensors and hip abductors of the participants. These muscle groups were chosen due to their importance for activities of daily living,^36–38^ and susceptibility to weakness and atrophy in hip osteoarthritis.^1,2,39^ NMES was applied unilaterally, to the affected limb of the participants with hip osteoarthritis, and to the right limb of the control group. For participants with bilateral hip osteoarthritis, NMES was applied to the more severely affected limb. To stimulate the knee extensors (the quadriceps femoris muscle group), two PALS platinum 70 mm (2.75”) round electrodes were positioned on the vastus lateralis and vastus medialis, in line with manufacturer instructions (Figure 1). For the hip abductors (gluteus medius, gluteus minimus and tensor faciae latae), two 70 mm round electrodes were placed over the proximal and distal components of the gluteus medius (Figure 2). Once the device was fitted, the participant operated the device independently for a period of around five minutes. Data were collected on device acceptability, tolerability and muscle contractile force and compared between groups to observe any differences in response to NMES in participants with hip osteoarthritis and healthy, age-matched controls.

### Variables

Age, weight, height and medical history were recorded from all participants. Affected side(s), duration of symptoms and the use of analgesia for pain relief were recorded from the participants in the hip osteoarthritis group. The subjective severity of hip pain when weight bearing was rated using the Numeric Pain Rating (NPR) scale (range 0–10 with 0 depicting no pain and 10 representing unbearable pain) and the severity of symptoms were quantified using the Oxford Hip Score.^33^

#### Tolerability

Once the device was fitted, participants independently operated the device and were instructed to gradually increase the current intensity, starting at 10 mA, until a visible involuntary muscle contraction was produced. If it was not possible to produce an involuntary muscle contraction, the participant was asked to increase the current intensity to the maximum tolerated for a period of around five minutes. Each mark on the stimulator corresponded to approximately 10 mA. The current intensity required to elicit an involuntary muscle contraction, or maximum current intensity tolerated, was recorded as a measure of device tolerability.

#### Pain and discomfort

Pain and discomfort were also used as measures of device tolerability. Pain during muscle contraction was recorded using a Numeric Rating Scale (NRS), with a score of zero describing no pain at all, and a score of 10 depicting the worst pain imaginable. If no visible muscle contraction was elicited, pain was recorded during the maximum stimulation intensity tolerated by the participant. Discomfort was assessed through the administration of a Likert Scale questionnaire that has previously been used to quantify discomfort associated with NMES.^40,41^ Participants were asked to score their discomfort in comparison to a blood pressure cuff inflated on the arm on a scale of one to five, with a score one depicting no discomfort and a score of five describing severe discomfort.

#### Muscle contractile force

To evaluate if the current intensity tolerated was sufficient to evoke an involuntary muscle contraction, and the relative feasibility of the device within rehabilitation, the strength of muscle contraction produced by NMES was scored through visual inspection and the definitions used in the Medical Research Council’s scale (MRC scale) of muscle power.^42^ Although it does not measure strength itself, the MRC scale is the most commonly accepted method of evaluating volitional muscle activation and has proven to be reliable and accurate for clinical assessment in weak muscles.^43^

Once an involuntary muscle contraction was produced, or the participant had reached the maximum intensity of stimulation tolerable, the muscle contraction was graded independently by one researcher using the descriptions in Table 1. For example, if the NMES device could not activate a muscle contraction (no trace or flicker), the investigator would award a score of zero. If a flicker or trace of muscle activation was observed, a score of one was awarded. During knee extensor stimulation, the participant was seated on the end of a plinth, other than during the assessment of MRC grade 2. For this assessment, the participant was side lying with their leg supported. For hip abduction, the participant was side lying, with their test side up.

**Table 1.** MRC scale of muscle power, used with permission of the Medical Research Council.

Score	Description
0	No muscle activation
1	Trace muscle activation, such as a twitch, without achieving full range of motion
2	Muscle activation without gravity resistance, achieving full range of motion
3	Muscle activation against gravity, full range of motion
4	Muscle activation against some resistance, full range of motion
5	Muscle activation against examiner’s full resistance, full range of motion

#### Acceptability

At the end of the testing session, participants were asked if they would consider using the device in a treatment routine (yes/no answer), and to provide any other comments or opinions about the NMES device.

### Sample size and statistical methods

A formal sample size calculation was not considered appropriate given the study design.^44^ Following recommendations for the design of usability studies in medical devices,^45^ a sample size of 15 participants per group was sought. Data were compared between groups to observe any differences in response to NMES that may be a result of hip joint pathology.

All data were analysed using IBM SPSS Statistics version 26 (SPSS Inc., Chicago, USA), with the significance level set at *p* < 0.05. Normality of the numerical data were analysed using a Shapiro-Wilk test. If both samples passed the preliminary normality test, an independent samples *t* test was conducted.^46^ The current intensity data were not normally distributed, and hence, a Mann-Whitney *U* test was conducted to compare tolerability between groups. Mean (standard deviation) and median (interquartile range (IQR)) were used to describe normally and non-normally distributed data, respectively.^47^ Categorical data were analysed using a Fisher’s exact test (two variables) or a Pearson’s chi-squared (more than two variables) and results were presented as percentages. Participant feedback on acceptability was categorised into key themes and reported using a descriptive analysis.

## Results

**Figure 3.**
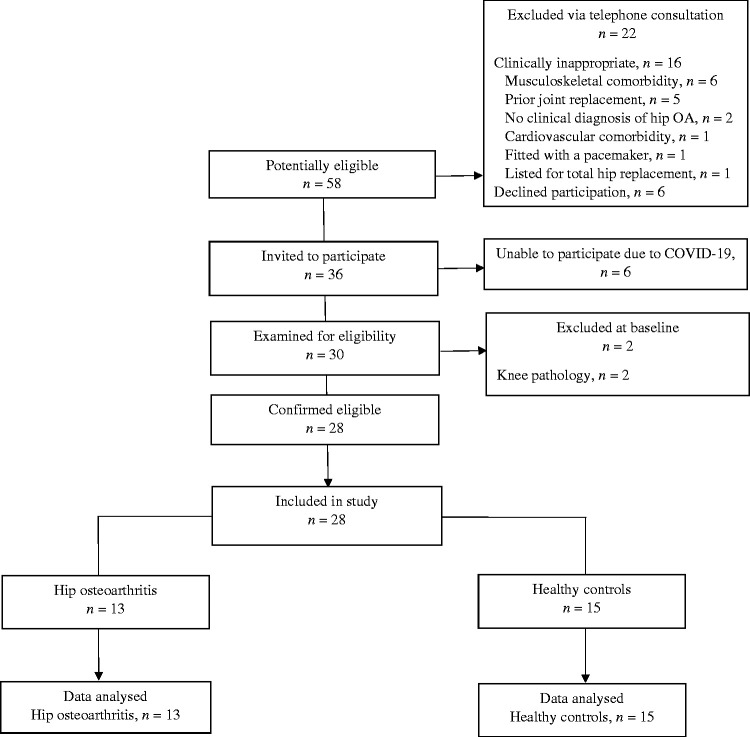
Participant recruitment through the study.

Fifty-eight individuals volunteered to take part in the study (Figure 3). During the initial telephone consultation, 16 volunteers did not meet the inclusion criteria due to: musculoskeletal comorbidity (*n* = 6); prior joint replacement (*n* = 5); hip pain but no clinical diagnosis of osteoarthritis (*n* = 2); cardiovascular comorbidity (*n* = 1), fitted with a pacemaker (*n* = 1); and listed for total hip replacement surgery (*n* = 1), and were excluded from the study. Six participants declined participation due to travel or time commitments. A total of 36 were invited to attend the testing session. Two participants in the control group were excluded during the eligibility assessment due to knee pathology not previously disclosed. A further six participants were unable to attend the testing session due to the COVID-19 pandemic and the Government advice to close higher education institutes. Hence, the study was prematurely closed on 15th March 2020. This analysis includes 28 participants who were recruited prior to the pandemic (hip osteoarthritis, *n* = 13; control group, *n* = 15).

There were no differences between groups in terms of age (*p* = 0.39) or gender distribution (*p* = 1.00). The hip osteoarthritis group had a significantly higher BMI than the control group (*p* = 0.03). Participants with hip osteoarthritis group had a mean Oxford Hip Score of 28 ± 7.81 (range: 18–39), suggesting moderate-to-severe hip osteoarthritis.^33^ The mean duration of symptoms was 4.04 ± 3.17 years (range: 6 months–10 years) and mean VAS pain on weight bearing was 5.31 ± 1.49 (range 3–8) (Table 2). Six participants were not taking any analgesics, four were taking paracetamol or ibuprofen when required, one was taking codeine and paracetamol, one was taking the maximum dose of paracetamol, and one participant was taking diahydrocodine in addition to cod liver oil.

**Table 2.** Characteristics of participants.

Characteristic	Unilateral hip OA *n* = 11	Bilateral hip OA *n* = 2	All hip OA *n* = 13	Control group *n* = 15
Age (years)	75 ± 7.69	72 ± 4.95	75 ± 7.30	72 ± 6.42
Males, n (%)	4 (36%)	1 (50%)	5 (38%)	5 (33%)
Height (m)	1.68 ± 0.08	1.70 ± 9.90	1.68 ± 0.08	1.68 ± 0.12
Weight (kg)	83.0 ± 18.29	91.00 ± 4.24	84.23 ± 17.01	71.85 ± 14.89
BMI (kg/m^2^)	29 ± 6	32 ± 2	30 ± 6	25 ± 4
Oxford Hip Score	27 ± 7	34 ± 5	28 ± 7	N/A
Pain (VAS)	5.79 ± 1.62	5.5 ± 0.71	5.31 ± 1.49	N/A
Duration of symptoms (years)	3.68 ± 2.82	6.0 ± 5.66	4.04 ± 3.17	N/A

### Tolerability

All participants were comfortable with the NMES sensation and tolerated electrical stimulation of the knee extensors and hip abductors for the testing period. The median current intensity tolerated during knee extensor stimulation in the osteoarthritis group was 45 mA (IQR: 40–50), and 47 mA (IQR 40–50) in the control group. The median current intensity tolerated during hip abductor stimulation in the osteoarthritis group was 45 mA (IQR: 40–50) and 40 mA (IQR: 40–50) in the control group. Self-selected maximum stimulation intensity did not differ between groups during electrical stimulation of the knee extensors (*p* = 0.89) or hip abductors (*p* = 0.45).

### Pain and discomfort

Pain during electrical stimulation was reported by one participant from each group during stimulation of the knee extensors. Pain was scored as 1/10 by the participant with osteoarthritis, and 4/10 by the participant in the control group. Pain during electrical stimulation of the hip abductors was reported by four participants (31%) in the osteoarthritis group (range: 2–7), and by three participants (20%) in the control group (range: 3–7). No discomfort was reported by the osteoarthritis group during stimulation of the knee extensors. Discomfort was more commonly reported during stimulation of the hip abductors (Table 3). There were no differences in discomfort between the osteoarthritis and control group during electrical stimulation of the knee extensors (*p* = 0.13) or hip abductors (*p* = 0.72).

**Table 3.** Discomfort experienced during electrical stimulation of the knee extensors and hip abductors in adults with hip osteoarthritis, compared to healthy older adults.

	Knee extensors			Hip abductors		
Discomfort	Osteoarthritis	Control	Sig (2-tailed)	Osteoarthritis	Control	Sig (2-tailed)
Minimal discomfort	13 (100%)	11 (73%)	*p* = 0.13	8 (62%)	11 (73%)	*p* = 0.72
Mild discomfort	0	3 (20%)		2 (15%)	1 (7%)	
Moderate discomfort	0	1 (7%)		3 (23%)	3 (20%)	

### Muscle contractile force

Neuromuscular electrical stimulation of the knee extensors evoked an involuntary muscular contraction in 11 participants (85%) in the hip osteoarthritis group and 15 participants (100%) in the control group, at a stimulation intensity acceptable to the participant. Electrical stimulation of the hip abductors evoked an involuntary muscular contraction in eight participants (62%) in the osteoarthritis group, and ten participants (67%) in the control group. Muscle contractile force, as measured by the MRC scale for muscle power, was not significantly different between study groups during stimulation of the knee extensors (*p* = 0.29) or hip abductors (*p* = 1.00). However, muscle contractile force was greater in the knee extensors, when compared to the hip abductors, in both study groups (Table 4).

**Table 4.** Muscle contractile force during unilateral electrical stimulation of the knee extensors and hip abductors in adults with hip osteoarthritis, compared to healthy older adults.

	Knee extensors			Hip abductors		
MRC grade	Osteoarthritis	Control	Sig (2-tailed)	Osteoarthritis	Control	Sig (2-tailed)
0 No muscle activation	2 (15%)	0	*p* = 0.29	5 (39%)	5 (33%)	*p* = 1.00
1 Trace muscle activation	1 (8%)	4 (27%)		8 (62%)	10 (67%)	
2 Activation without gravity resistance	9 (69%)	10 (67%)		0	0	
3 Activation against gravity	1 (8%)	1 (7%)		0	0	

### Acceptability

All participants in both study groups reported that they would consider using electrical stimulation of the knee extensors and hip abductors in a treatment routine. Two participants in the osteoarthritis group and two in the control group expressed concern with the process of independently locating the muscles and placing electrodes. Two participants in the osteoarthritis group reported pain relief during stimulation of the hip abductors. Five participants in the control group said they would not have been able to tolerate a current higher than their self-selected maximum. One participant in the control group referred to the device as distracting rather than uncomfortable, and one described it as a useful alternative or adjunct to conventional exercise.

## Discussion

Electrical muscle stimulation has a long-established place in therapy practice^48^ and has been shown to preserve or restore muscle mass and aspects of neuromuscular function in a range of musculoskeletal conditions, including both acute injuries and chronic conditions.^23^ Nonetheless, NMES therapy remains clinically underutilised in the hip osteoarthritis population.^30^ The slow transition of NMES into clinical practice has been attributed to a lack of guidelines on stimulation parameters, uncertainty regarding the feasibility of stimulation for inducing strength gains, and concerns of intolerance in patients particularly sensitive to electrical stimulation.^22^ A key component of assessing the feasibility of clinical interventions is patient acceptability, which relates to how the intended recipients react to the intervention.^49^ In this preliminary study, the feasibility and acceptability of the NMES device were measured in a cohort of participants with advanced hip osteoarthritis, and compared to a cohort of healthy, age-matched controls, to observe any differences in stimulation response attributable to hip joint pathology.

Neuromuscular electrical stimulation of the knee extensors elicited a visible muscular contraction in 11 participants (85%) in the hip osteoarthritis group and 15 participants (100%) in the control group, at a stimulation intensity acceptable to the participant. Electrical stimulation of the hip abductors elicited a muscular contraction in eight participants (62%) in the osteoarthritis group, and ten participants (67%) in the control group. Muscle contractile force, pain, discomfort and acceptability did not differ between groups, however electrical stimulation of the knee extensors was favoured across all measures of assessment when compared to the hip abductors in both groups. These findings suggest that electrical stimulation of the knee extensors may be an efficacious and acceptable treatment modality to address muscle weakness in the hip osteoarthritis population. These findings are perhaps not surprising, given the evidence for NMES alone or combined with exercise for quadriceps strengthening in patients with osteoarthritis of the knee,^50^ but nonetheless provide important information for future research endeavours in this area.

Importantly, no differences were observed in muscle contractile force between the two study groups during stimulation of the knee extensors or hip abductors. NMES involves the application of electrical impulses to skeletal muscles, by means of surface electrodes placed over the muscle belly, with the ultimate goal to evoke visible muscle contractions.^22^ The basic theoretical premise of electrical muscle stimulation is that if the peripheral nerve can be stimulated, the resulting excitation impulse will be transmitted along the nerve to the motor endplates in the muscle, producing a muscle contraction, which will have an eventual effect on muscle hypertrophy and strength.^51^ Aerobic exercise and local muscle strengthening are recommended as core components in the management of hip osteoarthritis,^9–11^ however, voluntary exercise may be inhibited by pain during joint loading. During electrical stimulation of the knee extensors, it was possible to achieve muscle activation and full range of motion in the majority of participants, with only two reports of pain. Clinically, these findings are important for patients who cannot perform conventional, voluntary exercise at either sufficient intensity or duration to be effective.

Interestingly, it was not possible to achieve a muscle contraction at a tolerable level of stimulation of the hip abductors in over one third of each study group, and the most powerful contraction elicited, as graded by the MRC scale, was a trace muscle activation. These findings may be explained by a higher percentage of fatty infiltration in the gluteal muscles when compared to the quadriceps and the substantial decrease in contractile tissues of the gluteal muscles evident in patients with hip osteoarthritis.^52–55^ Due to the high resistivity of subcutaneous fat tissue, higher stimulus currents are required to evoke muscle contractions where there is higher skeletal muscle fat infiltration, which can lead to patient discomfort.^56^ These predictions are supported by the assessment of tolerability, whereby both pain and discomfort were more frequently reported in both study groups during electrical stimulation of the hip abductors when compared to the knee extensors. From these findings, we can anticipate that electrical stimulation of the knee extensors will be more acceptable than electrical stimulation of the hip abductors in the hip osteoarthritis population. These findings are promising given the success of NMES applied to the knee extensors in individuals with knee osteoarthritis, whereby electrical stimulation has been shown to increase strength, train endurance, minimise atrophy, reduce pain and increase range of motion.^23,57^ Future research is required to examine the effectiveness of NMES for improving knee extensor strength and endurance in the hip osteoarthritis population.

## Limitations

A clear limitation of this study is the failure to meet the sample size sought due to a global pandemic and the premature completion of data collection. Participants were encouraged to answer the questions on the NMES device honestly and accurately. Nonetheless, we recognise an element of response bias may exist in the feedback of the device, whereby the participants felt they should report a favourable opinion.^58^ It should be acknowledged that the size of the electrode used with electrical stimulation can markedly affect the stimulation response, and that choosing a larger electrode may have improved the strength of contraction. In addition, tolerance to stimulation can increase with repeated use,^59^ and thus a higher current intensity may be achieved over time. The continuous contraction length used in this study may be less comfortable than the intermittent stimulation used most with NMES. Finally, the MRC grade is a subjective measure, and only quantifies the category of contraction strength, not strength itself.^43^

## Conclusions

Neuromuscular electrical stimulation of the knee extensors may be a feasible treatment method to address muscle weakness in the hip osteoarthritis population. NMES was well-tolerated and acceptable to participants and may serve as an alternative or adjunct treatment to improve muscle function for those who have difficulty participating in voluntary exercise. Future research evaluating the effectiveness of NMES for improving strength, endurance or minimising atrophy is required to progress these findings.
